# Structure of the Exopolysaccharide Secreted by a Marine Strain *Vibrio alginolyticus*

**DOI:** 10.3390/md16050164

**Published:** 2018-05-15

**Authors:** Sophie Drouillard, Isabelle Jeacomine, Laurine Buon, Claire Boisset, Anthony Courtois, Bertrand Thollas, Pierre-Yves Morvan, Romuald Vallée, William Helbert

**Affiliations:** 1 Université Grenoble Alpes, CERMAV, CNRS, Centre de Recherches sur les Macromolécules Végétales (CERMAV), BP 53, 38041 Grenoble CEDEX 9, France; sophie.drouillard@cermav.cnrs.fr (S.D.); isabelle.jeacomine@cermav.cnrs.fr (I.J.); laurine.buon.drouillard@cermav.cnrs.fr (L.B.); claire.boisset-helbert@cermav.cnrs.fr (C.B.); 2 Polymaris Biotechnology, Aéropôle Centre, 29600 Morlaix, France; Anthony.courtois@polymaris.com (A.C.); Bertrand.thollas@polymaris.com (B.T.); 3 Codif International, 35400 Roz-sur-Couesnon, France; py.morvan@codif.com (P.-Y.M.); r.vallee@codif.com (R.V.)

**Keywords:** *Vibrio alginolyticus*, nosturonic acid, cosmetic

## Abstract

*Vibrio alginolyticus* (CNCM I-4151) secretes an exopolysaccharide whose carbohydrate backbone is decorated with amino acids, likely conferring its properties that are appreciated in cosmetics. Here, the secreted polysaccharide of another strain of *V. alginolyticus* (CNCM I-5034) was characterized by chromatography and one- and two-dimensional NMR spectroscopy experiments. The structure was resolved and shows that the carbohydrate backbone is made of four residues: D-galactose (Gal), D-galacturonic acid (GalA) D-N-acetylglucosamine (GlcNAc) and D-glucuronic acid (GlcA), forming a tetrasaccharide repetition unit [→4)-β-d-GlcA-(1→3)-α-d-Gal-(1→3)-α-d-GalA-(1→3)-β-GlcNAc(1→]. GlcA is derivatized with a lactate group giving ‘nosturonic acid’, and GalA is decorated with the amino acid alanine.

## 1. Introduction

Polysaccharides are the most abundant and most diverse biopolymers on land and in the ocean. The wide stereochemical variability of monosaccharides and the numerous linkage possibilities offer an immense number of possible polysaccharide structures. For example, one estimation indicates that there are likely more than 10^12^ possible combinations to make a reducing hexasaccharide [1]. Another level of complexity stems from the decoration of the carbohydrate backbone with organic (e.g., acetate, pyruvate) or inorganic (e.g., sulfate ester) derivatives [2]. The diversity of chemical structures that are encountered in nature is correlated with the large diversity of three-dimensional conformations, leading to a large variety of networks (e.g., fibers, gels) having physico-chemical properties that meet the needs of specific industries.

Many bacteria, including marine bacteria, secrete extracellular polysaccharides, called exopolysaccharides (EPSs). The structural diversity of EPSs—which is largely underestimated—constitutes an immense portfolio of novel molecules. Marine EPSs have aroused considerable interest, and in vitro experiments highlight their biological activity, including anti-tumor activity, immunostimulatory activity, and anticomplementary activity, as well as the involvement in bone and tissue regeneration [3,4,5]. However, their use in industry as gelling or thickening agents faces heavy competition from the already marketed terrestrial bacterial EPSs, such as xanthan (*Xanthomonas campestris*) or gellan (*Sphingomonas paucimobilis*), or plant and algal polysaccharides. The high production costs of marine EPSs and compliance with regulations that are associated to the introduction of novel ingredient on the market (e.g., demonstration of safety) make industrial-scale development presently insurmountable [6].

The potential biological activity of marine EPSs combined with their interesting rheological properties make them very attractive for niche applications in the biomedical and cosmetics sectors. The production of marine EPSs also has several technical advantages. Large-scale production can be easily controlled, and, in contrast to that of plant and algal EPSs, is independent of seasonal variation. Due to their very high molecular weight, EPSs can be easily separated from other molecules and lend themselves to high-degree purification. A recent study shows that EPSs secreted by the Gram-negative marine bacteria *Vibrio alginolyticus* (CNCM I-4994) is decorated by amino acids, which are probably responsible for the biological properties of the macromolecules that are used as an ingredient in the cosmetics industry under the trademark “EPS White” [7]. Here, we report the structure of the EPS secreted by another marine Gram-negative *V. Galactose alginolyticus* strain (CNCM I-5034), which is also decorated with an amino acid residue and a rare “nosturonic acid” residue. Based on preliminary results, this polysaccharide has promising wound healing properties and stimulate skin immunity. 

## 2. Results and Discussion

### 2.1. Composition of the Vibrio alginolyticus Exopolysaccharide

Bradford assays did not detect proteins in the purified *V. alginolyticus* EPS (VA-EPS), attesting to the high purity of the sample. After complete hydrolysis of the polysaccharide, high-performance liquid chromatography detected alanine, suggesting that it was not a free amino acid. Size-exclusion chromatography (SEC), coupled to multi-angle laser light scattering confirmed that the purified EPS is likely composed of one species of molecule having a molecular weight (MW) of 1.11 × 10^6^ Da with a narrow polydispersity index of 1.2.

Composition analysis using gas chromatography (GC), after the complete hydrolysis of the polysaccharide and derivatization of the products, revealed galactose (Gal), galacturonic acid (GalA), and glucosamine (GlcN). Other signals that are present in the gas chromatogram had retention times that differed from the standard residues that were available in the laboratory and could not be attributed at this stage. Elemental analysis did not uncover any inorganic derivatives, such as sulfate or phosphate ester groups. Methylation analyses that are presented in Table 1 showed that Gal and GlcN are involved in 1,3-linkages and the GalA is linked at position 3 and 6.

**Figure 1:**
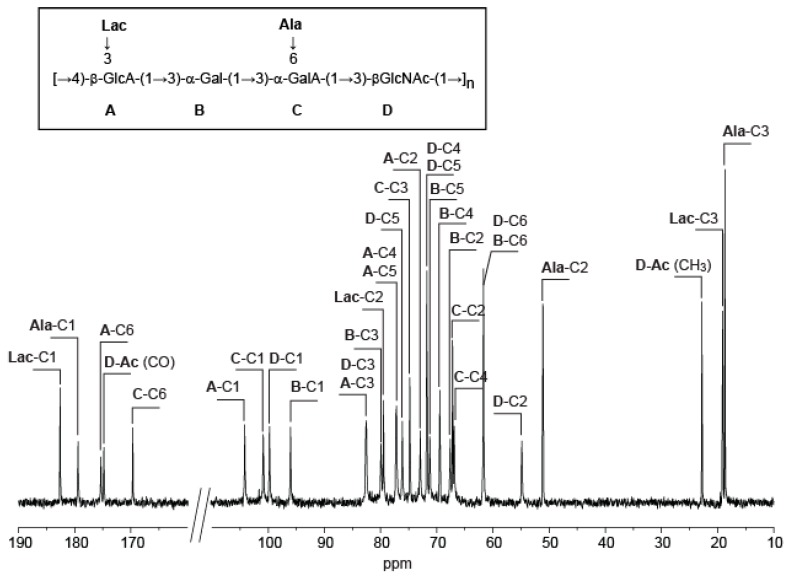
^13^C NMR of the *Vibrio alginolyticus* exopolysaccharide that was recorded at 343 K. Inset: chemical structure of the polysaccharide. GlcA: D-glucuronic acid, Gal: D-galactose, GalA: D-galacturonic acid, GlcNac: N-acetyl-D-glucosamine, Lac: lactate, Ala: alanine.

The ^13^C NMR spectrum of the polysaccharide (Figure 1) showed four signals at 95–105 ppm that were attributed to four anomeric carbons. The three signals that were observed in the region of the methyl groups (15–25 ppm) were attributed to the methyl group of an acetyl group (22.81 ppm), alanine (18.77 ppm), and lactate (19.09 ppm). Five signals were observed in the region of the carbonyl groups between 170 and 185 ppm and likely correspond to C6 of two uronic residues, the carboxyl groups of the amino acid and GlcNAc, and lactate.

**Figure 2:**
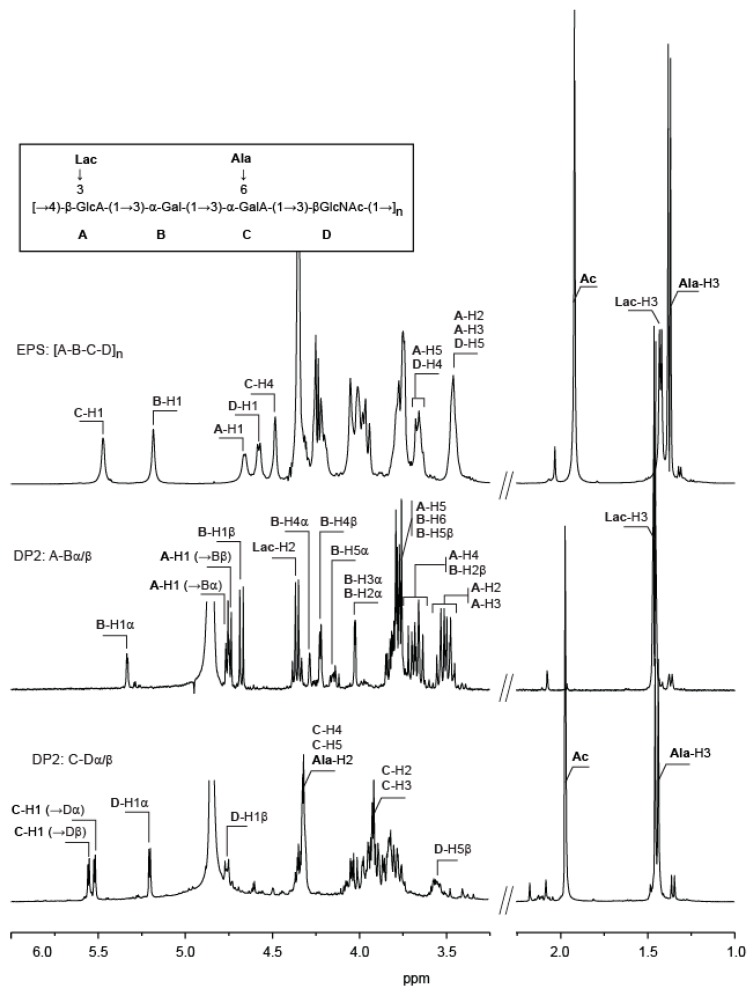
^1^H NMR of the *Vibrio alginolyticus* exopolysaccharide and the two disaccharides purified after acid hydrolysis. A: The ^1^H NMR of the polysaccharide was recorded at 343 K. B and C: ^1^H NMR of the A, B and C, D disaccharides, respectively, recorded at 293 K. Inset: chemical structure of the polysaccharide.

^1^H NMR spectra (Figure 2), as well as ^1^H/^13^C heteronuclear single-quantum correlations (HSQC) of the VA-EPS (supplementary Figure S1), confirmed a repetition unit made of four distinct residues. Integration of anomeric residues indicated an equimolar abundancy of the four residues. Two residues (B and C) adopt α-anomeric configuration and the two others (A and D) have a β-anomeric configuration. ^1^H NMR spectra also confirmed that the polysaccharide backbone is decorated with N-acetyl, alanine, and lactate groups.

The ring protons of the two α-linked residues (B and C) were assigned, starting from the anomeric protons B-H1 and C-H1 by successfully combining correlation spectroscopy (COSY, supplementary Figure S2) and total COSY (TOCSY) analyses (Table 2). The chemical shifts of the corresponding carbons were determined using HSQC and are reported in Table 3. The chemical shifts of residues B and C are in agreement with a α-d-galactose and α-d-galacturonic acid residues, respectively. Notably, the C-H4 protons that were observed at about 4.50 ppm, which showed weak *J*^3,4^ and *J*^4,5^ coupling constants, are characteristic of α-d-galacturonic acid. The correlation between the carbons C-C6 of the carboxyl group (169.55 ppm) with the proton of the amino acid Ala-H2 (4.24 ppm) was visible in heteronuclear multi-bond correlations (HMBC, supplementary Figure S3), indicating that alanine is linked to the GalA residue via an amide linkage. Integration of GalA and alanine protons showed that all GalA were decorated with the amino acid, and that no free GalA residues were present.

Assignment of the protons and the carbons of residue D was conducted similarly using COSY, TOCSY, and HSQC analyses. The chemical shifts reported in Table 2 and Table 3 are in agreement with a fully acetylated β-GlcN (β-GlcNAc) residue with the characteristic D-H2 proton and D-C2 carbon recorded at 3.79 ppm and 54.86 ppm, respectively. In HMBC (supplementary Figure S3), the carbon D-Ac (CO: 174.81 ppm) of the acetyl group correlated with the proton D-H2 (3.79 ppm) and the proton of the acetyl group (1.92 ppm). Deacetylated GlcN with H2 proton expected at around 2.7 ppm was not observed.

The carbon and proton chemical shifts measured for residue A did not show the characteristic values for Gal, GalA, and GlcNAc residues, as determined by composition analyses. The strong *J*^2,3^ coupling constant (observed on A-B oligosaccharide, see below) suggests that residue A belongs to the gluco or galacto series. The first attempts to determine the structure of residue A based on the spectra recorded on the polymer suggested a derivated glucuronic residue, with A-C6 (175.33 ppm) that was correlated with the A-H5 (3.67 ppm) and A-H4 (3.98 ppm) in HMBC.

### 2.2. Structural Analysis of the Two Hydrolysis Products of the V. alginolyticus Exopolysaccharide

**Figure 3:**
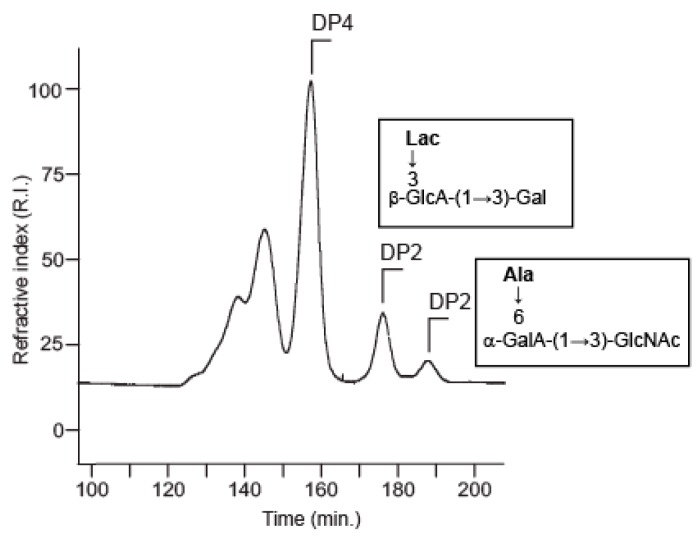
Size exclusion chromatography. Two disaccharides (structure in inset) were purified from the acid-hydrolyzed exopolysaccharides.

Mild acid hydrolysis of the polysaccharide (20 min in 1 M trifluoroacetic acid (TFA) at 100 °C) led to the production of a series of oligosaccharides that were fractionated using SEC (Figure 3). ^1^H NMR spectra of the two fractions that were collected at elution times of 178 min and 184 min are given in Figure 3. These spectra corresponded to two disaccharides, which were obtained pure. Other oligosaccharides of higher molecular weight were found in mixture and were not analyzed further.

The disaccharide eluting at 184 min were composed of residues C (GalA) and D (GlcNAc). Residue D was localized at the reducing end (D-H1α: 5.20 ppm and D-H1β: 4.75 ppm). Proton and carbon signals were ascribed straightforwardly while combining COSY, HMBC, and HSQC experiments (Supplementary Figures), and values that were reported in Table 2 and Table 3 are in agreement with a fully N-acetylated glucosamine. 

**Figure 4:**
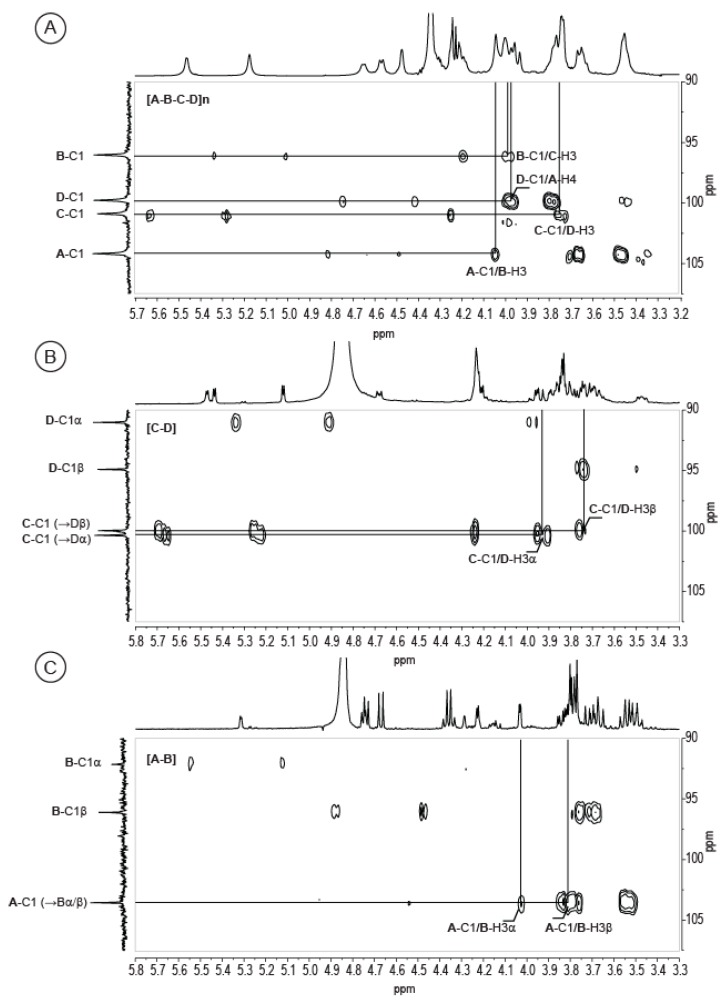
Detail of heteronuclear multi-bond correlations (HMBC) spectra. The spectra were recorded on the complete polysaccharide (**A**) and the two purified disaccharides (**B**,**C**). ^1^H/^13^C heteronuclear correlations that helped to determine linkages between residues are indicated in the spectra. The spectra were recorded at 343 K (**A**) and 293 K (**B**,**C**).

Similarly, NMR analyses confirmed that residue C is a galacturonic acid. The HMBC spectrum recorded on the C-D disaccharide (Figure 4B) was better resolved than that of the native polymer and was more clearly revealed the amide linkage between alanine and GalA. It also revealed the linkage between residues C and D. The correlation between carbon C-C1 (101.08/100.70 ppm) with the protons D-H3α (4.01 ppm) and D-H3β (3.82 ppm) demonstrated that the disaccharide is made of a D-GalA bound to a GlcNAc via an α(1,3) linkage.

The second disaccharide, which eluted at 178 min, was composed of a D-Gal residue (residue B) positioned at the reducing end (B-H1α: 5.33 ppm, B-H1β: 4.68 ppm). The chemical shifts of the carbon and proton signals were ascribed and are given in Table 2 and Table 3. Residue A was the second component of the disaccharide whose structure could not be elucidated based on the NMR spectra that were recorded on the polymer.

**Figure 5:**
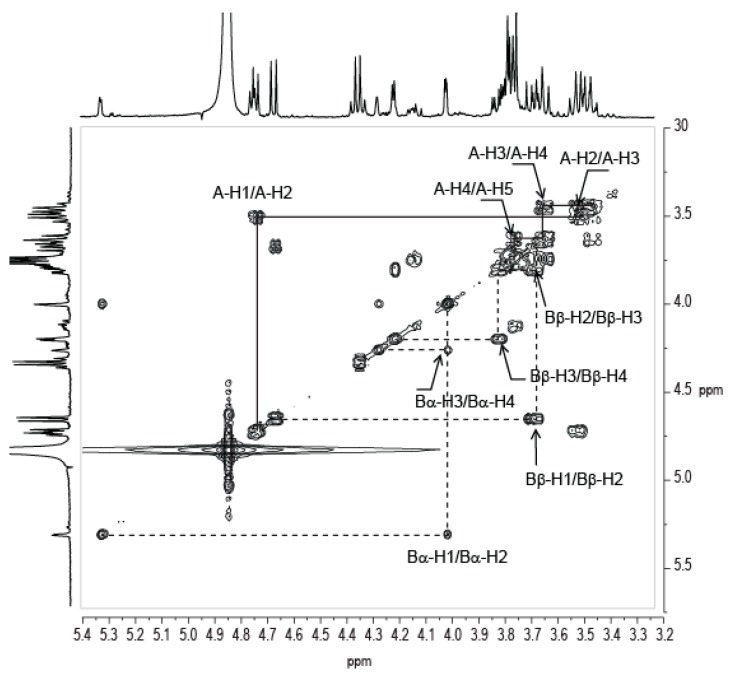
Correlation spectroscopy (COSY) spectrum of the A-B disaccharide [β-d-3LacGlcA-(1→3)-α/β-D-Gal]. The proton correlation system of the “nosturonic acid” residue is indicated. The spectra were recorded at 293 K.

The resolution of the NMR spectra of the A-B disaccharide made it possible to analyze residue A in detail. The proton system of residue A was determined using COSY (Figure 5) and TOCSY experiments. The measured coupling constants (*J*^1,2^ = 7.4 Hz, *J*^2,3^ = 13.9 Hz, *J*^3,4^ = 10.9 Hz, and *J*^4,5^ = 8.3 Hz) showed that the ring protons all have an equatorial conformation, demonstrating that residue A has a β-glucose ring structure. The coupling of A-H5 (3.77 ppm) with the carbon of the carboxylic group at A-C6 = 176.43 ppm suggests that the residue is a glucuronic acid residue. The measured chemical shifts are also in agreement with those that were expected for a glucuronic acid residue despite a slight shift of A-H3, which was observed at 3.48 ppm although usually being found around 3.55 ppm. Interestingly, we observed a coupling of lactate with residue A (Lac-C2 = 79.48 ppm correlated with A-H3 = 3.48 ppm in HMBC), which may explain the observed chemical shift. The lactate group linked to a glucuronic acid residue at position 3 has been observed in the EPS secreted by the cyanobacteria *Nostoc commune* DRH-1 [8]. The residue, named nosturonic acid (NosA) by the authors, has proton and carbons shift that are very similar to those that were observed in residue A. Therefore, we conclude that residue A has the same structure as the NosA residue. HMBC of the A-B disaccharide (Figure 4C) revealed a correlation of A-C1 (104.20 ppm) with proton B-H3α/β (4.02 ppm/3.82 ppm), demonstrating the 1,3 linkage between the two residues.

### 2.3. Complete Structure of the V. alginolyticus Exopolysaccharide

Analyses of the two disaccharides confirmed the polysaccharide composition that was deduced using gas chromatography experiments and NMR analyses, with a repetition moiety made of Gal, GalA, and GlcNAc. The structure of the fourth residue, 3Lac-GlcA (‘nosturonic acid’ residue), was deduced from analysis of the A-B [β-D-3LacGlcA-(1→3)-α/β-D-Gal] disaccharide. In addition, we were able to determine the linkage between the residues A and B, as well as the linkage between the residues C and D [α-D-6AlaGalA-(1→3)-α/β-D-GlcNAc]; both linkages were also observed in the HMBC spectrum of the complete polysaccharide. The correlations between A-C1 (104.20 ppm), B-H3 (4.05 ppm), C-C1 (100.90 ppm), and D-H3 (3.78 ppm) are highlighted in Figure 4A.

The D-A and B-C linkages absent in the studied oligosaccharides were observed in the HMBC spectrum given in Figure 4A. The D-C1 carbon (99.79 ppm) was correlated with the A-H4 proton (3.98 ppm), indicating that the β-d-GlcNAc (residue D) is linked to the β-D-3Lac-GlcA (residue A) via a β(1,4) linkage. Similarly, the correlation between B-C1 (96.04 ppm) and C-H3 (4.01 ppm) suggests that the linkage between the β-d-Gal (residue B) and the β-D-GalA (residue C) occurs through an α(1,3) linkage. Altogether, composition and NMR analyses corroborate, and indicate a polysaccharide structure made of a tetrasaccharide moiety decorated with one lactate and one alanine per repetition unit and whose formula is [→4)-β-d-3LacGlcA-(1→3)-α-d-Gal-(1→3) α-d-6AlaGalA-(1→3)-β-d-GlcNAc-(1→].

Decoration of the VA-polysaccharides backbone were very rarely encountered in bacteria, and, more especially, in *Vibrio* spp. Exploring the “carbohydrate structure database” (CSDB, http://csdb.glycoscience.ru/database/index.html, [9]), several structures of *Vibrio* sp. polysaccharides presented alanine derivatives, including *V. ordalii* [10], *V. cholera* [11], *V. parahaemolyticus* [12], *V. anguillarum* [13], and *V. alginolyticus* [14]. Similarly, *Vibrio spp* decorated by S-lactate were reported for strains of *V. cholerae* O144 and O76 strains [15,16], R-lactate in *V. fluvialis* [17] and uncharacterized lactate in *V. cholerae* O:1 [18]. However, no *Vibrio* polysaccharides that have characterized to date carried both alanine and R-/L-lactate derivatives confirming the very original structure of the VA polysaccharides investigated herein.

## 3. Materials and Methods

### 3.1. Production, Isolation and Purification of the Vibrio alginolyticus Exopolysaccharide

*V. alginolyticus* exopolysaccharide was produced by *Vibrio alginolyticus* (CNCM I-5034) in a 30 L fermenter containing marine broth medium (30 g/L sea salts, 1 g/L yeast extracts, 4 g/L peptone), which was supplemented with glucose (30 g/L) at 25 °C. The culture medium was inoculated at 10% (*v/v*) with a bacterial suspension in the exponential growth phase. The pH was adjusted and maintained at 7.2 by the automatic addition of 1 M NaOH. The medium was oxygenated at 15 L/min with an agitation rate of 350 rpm. After 72 h of fermentation, bacterial cells were removed from the culture medium by centrifugation (16,000 ×*g*, 30 min). The supernatant, containing the excreted VA-EPS, was then purified by filtration through a cellulose membrane (0.7 µm) and then by ultrafiltration (100 kDa). The filtration steps lead to a loss of 20 to 30% biomass giving a purified 1 g/L VA-EPS in water. The sample was freeze-dried and was stored at room temperature away from light and moisture.

### 3.2. Monosaccharide Analysis

The molar ratio of monosaccharides was determined according to [19], and was modified by [20]. The EPS was hydrolyzed with 3 M MeOH/HCl at 110 °C for 4 h, followed by re-N-acetylation with Ac2O overnight at room temperature. The methyl glycosides were converted to their corresponding trimethylsilyl derivatives. Separation and quantification of the per-O-trimethylsilyl methyl glycosides were performed using gas-liquid chromatography (GLC) on an Agilent system that was equipped with a HP-5 ms capillary column (Agilent 0.25 mm × 30 m). The trimethylsilyl derivatives were analyzed using the following temperature program: 120 °C for 1 min, 120 °C at 180 °C at 3 °C/min, 180 °C to 200 °C at 3 °C/min, 200 °C for 5 min.

### 3.3. Methylation Analysis

Glycosyl-linkage positions were determined as described in [21]. The native EPS was carboxyl-reduced by treatment with N-cyclohexyl 1-N′[β(N-methyl-morpholino)-ethyl] carbodiimide P-toluene sulfonate and with NaBD_4_ for 4 h at room temperature [22]. After dialysis against distilled water, the hydroxyl groups were methylated using 2.5 N butyl lithium in hexanes and methyl iodide in dimethyl sulfoxide (DMSO) [23]. The methylated compounds were extracted with CH_2_Cl_2_. The methylated products were then hydrolyzed in 2 M TFA for 2 h at 120 °C, then reduced with NaBD_4_ in a NH_4_OH solution for 30 min at 80 °C, and finally acetylated with 200 µL of 1-methyl imidazole and 2 mL of pyridine for 10 min at room temperature. Gaz chromatography-mass spectrometry (GC-MS) was performed on an Agilent instrument fitted with a high-performance 5 ms capillary column (Agilent, 0.25 mm × 30 m). The temperature program was 90 °C for 1 min, 90 °C to 300 °C at 5 °C/min, 300 °C for 1 min. Ionization was carried out in electron impact mode (EI, 70 eV).

### 3.4. Amino Acid Composition

*V. alginolyticus* exopolysaccharide (1% *w/v*) in water was hydrolyzed by addition of an equal volume of 11 N HCl for 5 h at 105 °C. After a cooling step, neutralization was carried out with 10 N NaOH, and then with 1 N NaOH, using pH-indicator paper to reach pH 6–7. The final volume was adjusted to 25 mL with ultrapure water and filtered on a 0.2 µm sterilizing grade membrane.

The neutralized samples and an amino acid hydrolysate standard (Waters) were derivatized by adding 6-aminoquinolyl-N-hydroxysuccinimidyl carbamate using the AccQ-Ta Ultra Derivatization kit (Waters). The derivatized samples (10 µL) were injected on a AccQ-Tag Ultra C18 column (1.7 µm 2.1 × 100 mm, Waters) that was mounted on a Waters Acquity H class ultra-high-performance liquid chromatography apparatus that was equipped with UV detector working at 260 nm. 

A gradient elution was conducted for 10 min at 55 °C starting with 100% eluent A (acetonitrile/formic acid) to 100% of eluent B (acetonitrile). Signals were compared with derivatized amino acid standards. According to experimental conditions, serine and alanine eluted at 2.3 min and 4.4 min, respectively.

### 3.5. Determination of Absolute Configuration

Assignment of absolute configuration of monosaccharide residues were adapted from the method of Gerwig [24,25]. 2 mg of polysaccharide was dissolved in 500 µL of 4N TFA and maintained, in sealed glass tubes, for 4 h at 100 °C. After cooling, TFA was evaporated under a flux of nitrogen. 500 µL of (S)-(+)-2-butanol and a drop of 13N TFA were added to the dried sample and the glass tube hermetically closed were kept 8 h at 80 °C. Butanol and TFA were evaporated under nitrogen. Butylglycosides samples were then re-N-acetylated, converted to their corresponding trimethylsilyl derivatives, and analyzed by GC-MS, according to the protocol described in Section 3.2. for monosaccharide analysis.

### 3.6. Molecular Weight Determination

The molecular weight of VA-EPS was determined by high-performance size-exclusion chromatography (HPSEC) using an eighteen-angle light scattering detector, which was coupled with refractive index detection and specific refractive index increment dn/dc (DAWNTM HELEOS, Wyatt). Elution was performed on Shodex OHpak SB-805 HQ and OHpak SB-806 HQ placed in series (Phenomenex, exclusion limit <2.10^7^ g/mol) with 0.1 M NaNO_3_ as the eluent. To calculate the molecular mass, the dn/dc value used was 0.145 mL/g. The polydispersity index was calculated from the Mw/Mn ratio.

### 3.7. Acid Hydrolysis and Oligosaccharides Purification

*V. alginolyticus* exopolysaccharide underwent mild acid hydrolysis. 300 mg of polysaccharide was solubilized in 75 mL 1 M TFA and was heated at 100 °C for 20 min. After neutralization with 8 N NH_4_OH, the salts were precipitated with five volume of acetone. Salts were eliminated by centrifugation and the oligosaccharides that were present in water/acetone solution were recovered by evaporation. The resulting oligosaccharides (about 180 mg) were fractionated using SEC on a Toyopearl HW-40 (Tosoh, exclusion limit <10^4^ Da) with 0.1 M (NH_4_)_2_CO_3_ as the eluent.

### 3.8. Nuclear Magnetic Resonance

Carbon-13 and proton NMR spectra were recorded with a Bruker Avance 400 spectrometer operating at a frequency of 100.618 MHz for ^13^C and 400.13 MHz for ^1^H. Samples were solubilized in D_2_O at a temperature of 293 K for the oligosaccharides and 343 K or 353 K for the polysaccharide. Residual signal of the solvent was used as the internal standard: HOD at 4.85 ppm at 293 K and 4.35 ppm at 343 K. ^13^C spectra were recorded using 90° pulses, 20,000 Hz spectral width, 65,536 data points, 1.638 s acquisition time, 1 s relaxation delay, and between 8192 and 16,834 scans. Proton spectra were recorded with a 4006 Hz spectral width, 32,768 data points, 4.089 s acquisition times, 0.1 s relaxation delays, and 16 scans. The ^1^H and ^13^C-NMR assignments were based on ^1^H-^1^H homonuclear and ^1^H-^13^C heteronuclear correlation experiments (correlation spectroscopy, COSY; heteronuclear multiple-bond correlation, HMBC; heteronuclear single quantum correlation, HSQC). They were performed with a 4006 Hz spectral width, 2048 data points, 0.255 s acquisition time, 1 s relaxation delay; 32 to 512 scans were accumulated.

## Figures and Tables

**Table 1:** Methylation analysis of the *Vibrio alginolyticus* exopolysaccharide (VA-EPS) after carboxyl reduction.

Type of Sugar	Partially Methylated Alditol Acetate	Deduced Linkage
Galactose	1,5-Di-O-acetyl-1-deuterio-2,3,4,6-tetra-O-methyl-d-galactitol	Gal*p*-(1→
Galactose	1,3,5-Tri-O-acetyl-1-deuterio-2,4,6-tri-O-methyl-d-galactitol	→3)-Gal*p*-(1→
N-acetyl glucosamine	1,3,5-Tri-O-acetyl-2-(acetylmethylamino)-2-deoxy-1-deuterio-4,6-di-O-methyl-d-Glucitol	→3)-GlcNAc-(1→

**Table 2:** Proton chemical shifts (ppm) of the *Vibrio aginolyticus* exopolysaccharide (VA-EPS) and the two purified disaccharides. A, B, C, and D refer to the residues composing the repetition moieties of the polysaccharide. Chemical shifts of alanine and lactate are sensitive to pH. Data were recorded at pH 7. GlcA: D-glucuronic acid, Gal: D-galactose, GalA: D-galacturonic acid, GlcNac: N-acetyl-D-glucosamine, Lac: lactate, Ala: alanine.

	VA-EPS [ABCD]_n_	DP2 ABα/β	DP2 CDα/β
**A: 3Lac-GlcA**			
H1	4.66	4.75/4.74 ^a^	
H2	3.46	3.52	
H3	3.46	3.48	
H4	3.98	3.66	
H5	3.67	3.77	
Lac-H2	4.32	4.36	
Lac-H3	1.42	1.46	
**B: Gal**			
H1	5.18	5.33/4.68	
H2	4.05	4.02/3.69	
H3	4.05	4.02/3.82	
H4	4.22	4.28/4.22	
H5	4.20	4.15/3.76	
H6	3.74	3.77	
**C: 6Ala-GalA**			
H1	5.47		5.51/5.55 ^b^
H2	4.01		3.91/3.90 ^b^
H3	4.01		3.93
H4	4.48		4.32
H5	4.26		4.32
Ala-H2	4.24		4.31
Ala-H3	1.38		1.44
**D: GlcNAc**			
H1	4.58		5.20/4.75
H2	3.79		4.04/3.82
H3	3.78		4.01/3.82
H4	3.65		3.78/3.77
H5	3.46		3.96/3.55
H6	3.95		3.87/3.91
H6’	3.75		3.80/3.80
NHAc:CH_3_	1.92		1.97

^a^ Residue A is linked to the α- or β-anomer of residue B (A-H1→Bα/A-H1→Bβ). ^b^ Residue C is linked to the α- or β-anomer of residue D (C-H1→Dα/C-H1→Dβ C-H2→Dα/C-H2→Dβ).

**Table 3:** Carbon chemical shifts (ppm) of the *Vibrio aginolyticus* exopolysaccharide (VA-EPS) and the two purified disaccharides.

	VA-EPS [ABCD]_n_	DP2 ABα/β	DP2 CDα/β
**A: 3Lac-GlcA**			
C1	104.20	104.19	
C2	72.96	72.94	
C3	82.53	84.63	
C4	77.32	72.86	
C5	77.16	76.98	
C6	175.33	176.43	
Lac-C1	182.60	182.77	
Lac-C2	79.48	79.40	
Lac-C3	19.09	19.40	
**B: Gal**			
C1	96.04	92.75/96.72	
C2	67.61	67.90/71.45	
C3	79.93	80.26/83.39	
C4	69.45	69.51/68.95	
C5	71.25	70.84/75.48	
C6	61.68	61.63/61.82	
**C: 6AlaGalA**			
C1	100.90		101.08/100.70 ^a^
C2	67.20		68.58/68.74 ^a^
C3	74.80		69.55
C4	66.91		70.44/70.36 ^a^
C5	71.76		71.98/71.92 ^a^
C6	169.55		170.20/170.32 ^a^
Ala-C1	179.40		180.01
Ala-C2	51.11		51.25
Ala-C3	18.77		18.63
**D: GlcNAc**			
C1	99.79		91.76/95.64
C2	54.86		53.07/55.74
C3	82.65		79.92/81.46
C4	71.76		71.26/71.24
C5	76.13		71.85/76.18
C6/C6’	61.68		60.97/61.10
NHAc:CO	174.81		174.86/175.16
NHAc:CH_3_	22.81		22.44/22.70

^a^ Residue C is linked to α- or β-anomer of residue D (C-C1→Dα/C-C1→Dβ).

## Supplementary Materials

The following are available online at http://www.mdpi.com/1660-3397/16/5/164/s1.
