# Supplementary material for: Structure of the Exopolysaccharide Secreted by a Marine Strain Vibrio alginolyticus
Source: Mar Drugs. 2018 May 15;16(5):164. doi: 10.3390/md16050164 (PMC5983295; doi:10.3390/md16050164)
Supplement: Supplementary file 1 [file marinedrugs-16-00164-s001.zip › marinedrugs-249332-SI.pdf]

marine drugs

MDPI

Supplementary informations

Structure of the exopolysaccharide secreted by a marine strain *Vibrio alginolyticus*

Sophie Drouillard [1], Isabelle Jeacomine [1], Laurine Buon [1], Claire Boisset [1], Anthony Courtois [2], Bertrand Thollas [2], Pierre-Yves Morvan [3], Romuald Vallée [3] and William Helbert [1*]

[1] Université Grenoble Alpes, CERMAV, 38000 Grenoble, France
CNRS, Centre de Recherches sur les Macromolécules Végétales (CERMAV), 38000 Grenoble, France; E-Mails: sophie.drouillard@cermav.cnrs.fr (S.D.); isabelle.jeacomine@cermav.cnrs.fr (I.J.); laurine.buon.drouillard@cermav.cnrs.fr (L.B.); claire.boisset-helbert@cermav.cnrs.fr (C.B); william.helbert@cermav.cnrs.fr (W.H.)

[2] POLYMARIS BIOTECHNOLOGY, Aéropôle Centre, 29600 Morlaix; E-Mails: Anthony.courtois@polymaris.com (A.C.); Bertrand.thollas@polymaris.com (B.T.)

[3] CODIF INTERNATIONAL, 35400 Roz-sur-Couesnon, France; E-Mails: py.morvan@codif.com (P.M.); r.vallee@codif.com (R.V.)

* Correspondence: william.helbert@cermav.cnrs.fr (W.H.); Tel.: +33 (0)4 76 03 76; Fax: +33 (0)4 76 54 72 03.

```
Lac                               Ala
 ↓                                 ↓
 3                                 6
[→4)-β-GlcA-(1→3)-α-Gal-(1→3)-α-GalA-(1→3)-βGlcNAc-(1→]n
  A             B            C              D
```

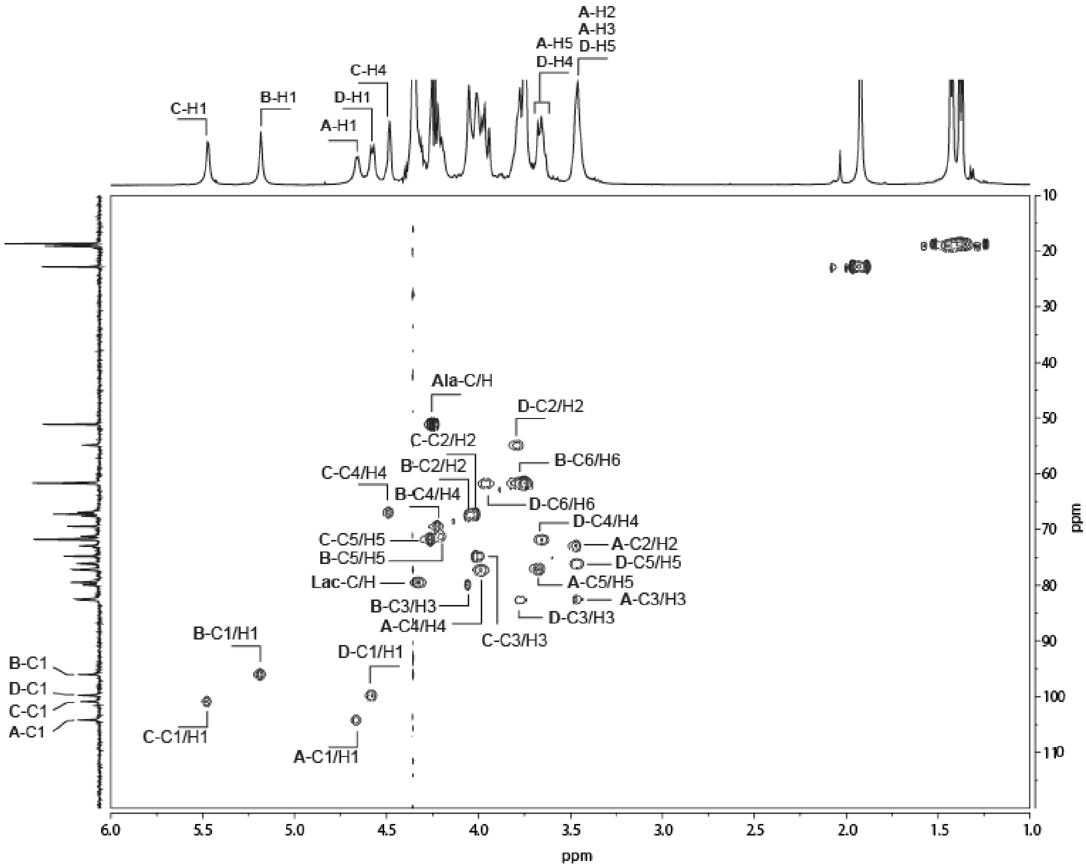

Figure S1. HSQC spectrum of the the *Vibrio alginolyticus* exopolysaccharide. The spectrum was recorded at 343 K.

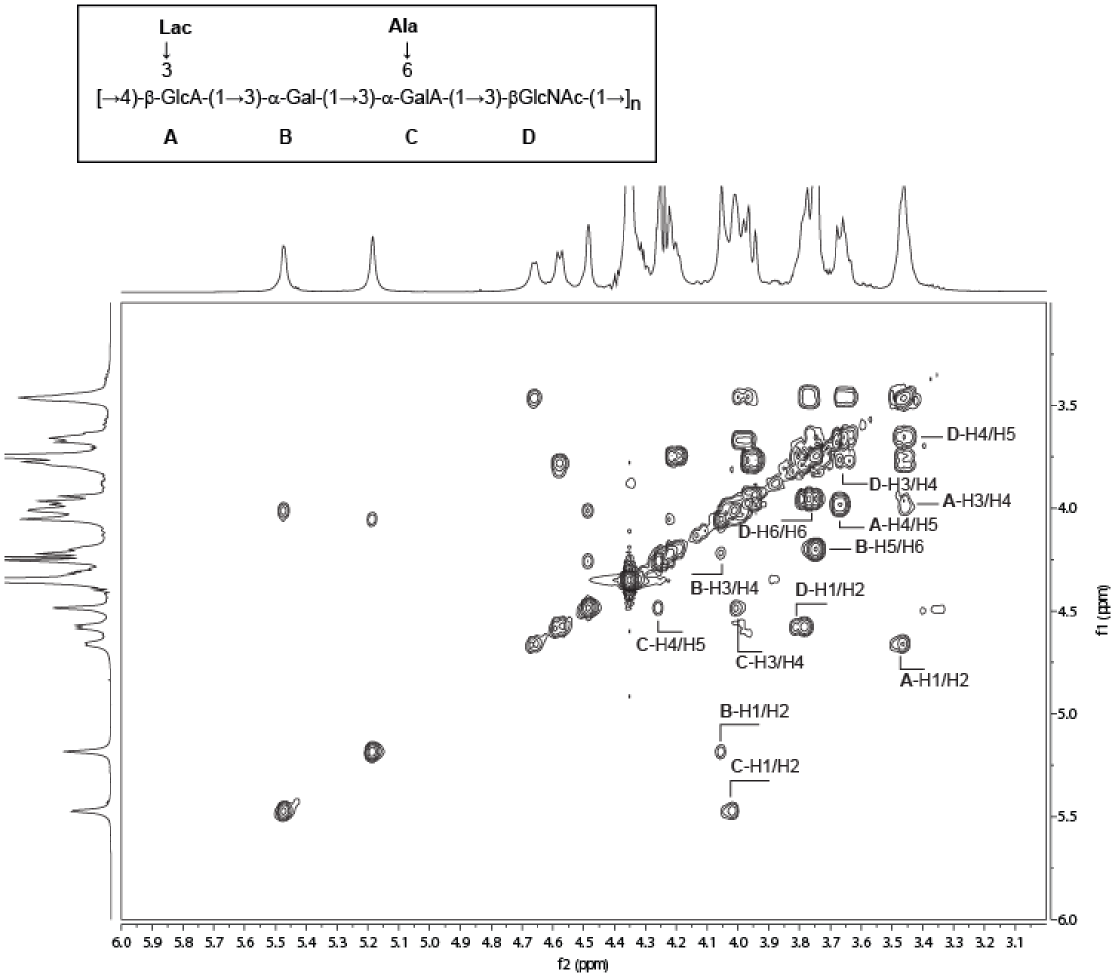

Figure S2. COSY spectrum of the *Vibrio alginolyticus* exopolysaccharide. The spectrum was recorded at 343 K.

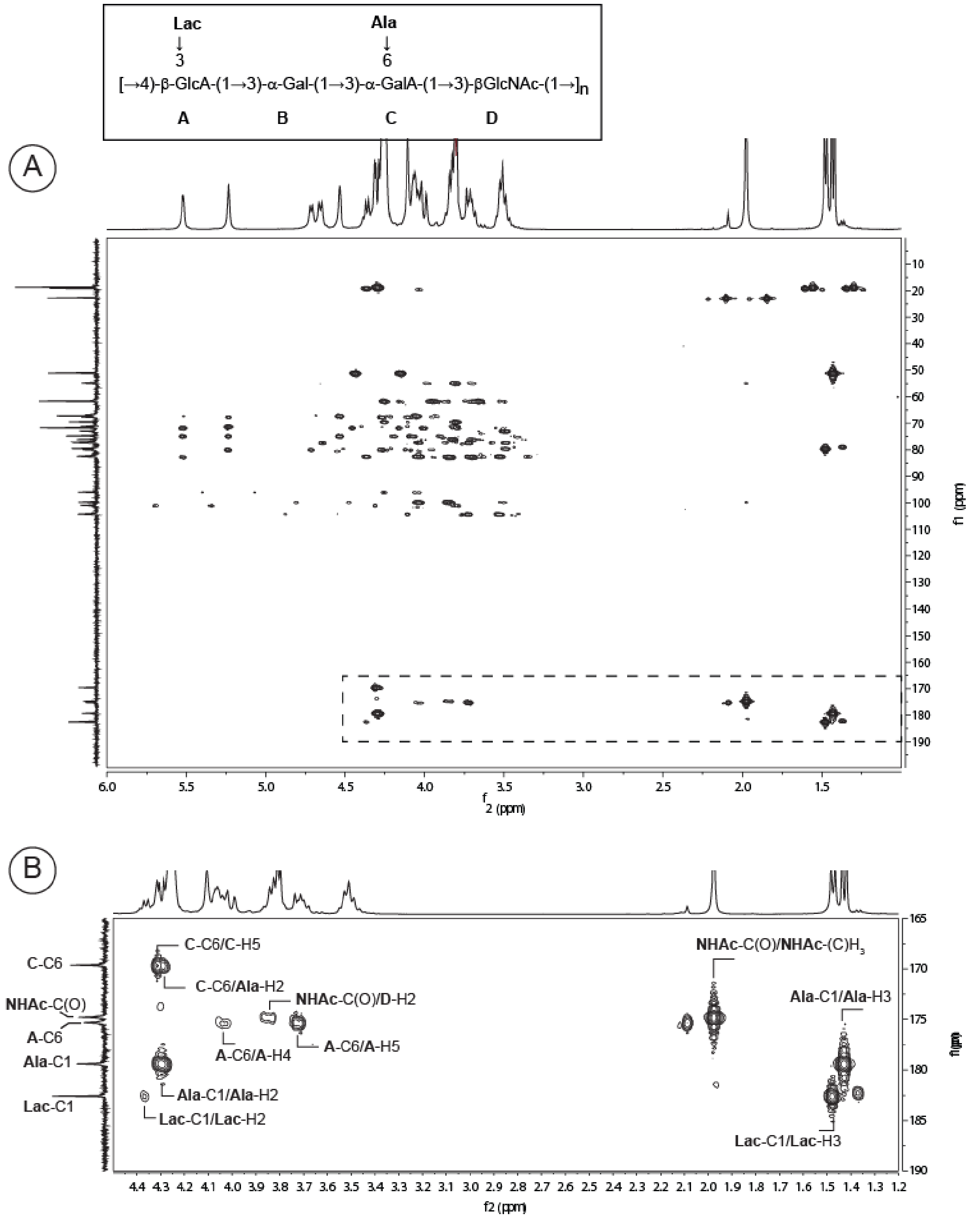

Figure S3. A) HMBC spectrum of the *Vibrio alginolyticus* exopolysaccharide. The spectrum was recorded at 353 K. **B**) Enlarged part of the HMBC spectra highlighting the coupling of alanine and lactate derivatives.
